# Quantitative analysis on the wear of monolithic zirconia crowns on antagonist teeth

**DOI:** 10.1186/s12903-021-01452-z

**Published:** 2021-03-04

**Authors:** Zhenyu Tang, Xinyi Zhao, Hui Wang

**Affiliations:** 1 State Key Laboratory of Military Stomatology & National Clinical Research Center for Oral Diseases & Shaanxi Key Laboratory of Oral Diseases, Department of Dental Materials, School of Stomatology, The Fourth Military Medical University, Xi’an, China; 2 Department of Stomatology, Liuzhou People’s Hospital, Liuzhou, China; 3 Guilin Medical University, Guilin, China

**Keywords:** Y-TZP ceramic, Monolithic zirconia crown, Enamel, Wear, Roughness, Three‐dimensional topography

## Abstract

**Background:**

The present study aimed to quantitate the wear of the highly transparent Yttria-stabilized tetragonal zirconia polycrystals (Y-TZP) ceramic monolithic zirconia crown on the enamel in vivo and discuss the prone position of the wear and the underlying mechanism.

**Methods:**

A total of 43 patients with 43 posterior teeth were selected for full zirconia crown restoration and examined immediately, at 6 months, and at 1 year after restoration. During the follow-up visit, the fine impression of the patients’ monolithic zirconia crowns, the antagonist teeth, the corresponding contralateral natural teeth, the super plaster cast, and epoxy resin model was ontained. The model of epoxy resin was observed under a stereo microscope, and the microstructure parts were observed under a scanning electron microscope.

**Results:**

After 1 year, the mean depth and volume of wearing of the monolithic zirconia crown were the smallest (all *P* < 0.01), while those of the antagonist teeth were significantly larger than those of the natural teeth (*P* < 0.0001), and no significant difference was found among the natural teeth (*P* = 0.3473, *P* = 0.6996). The amount of wear after one year was remarkably higher than that at 6 months (*P* < 0.0001). The microscopic observation revealed the tendency of wearing of the monolithic zirconia crown on the antagonist teeth at the protruding early contact points. Electron micrographs of tooth scars showed that the wearing mechanism of the monolithic zirconia crown on natural teeth was mainly abrasive and fatigue wear.

**Conclusions:**

Although the self-wearing is insignificant, the monolithic zirconia crown can cause wear of the antagonist teeth via occlusal or early contact significantly; the amount of wearing is higher than that of natural teeth and increases over time. The wearing mechanism is mainly abrasive and fatigue wear.

## Background

Yttria-stabilized tetragonal zirconia polycrystals (Y-TZP) ceramic has the mechanical characteristics of high strength, high hardness, and high fracture toughness [1, 2], rendering it as an ideal dental restoration material. The early Y-TZP ceramic had low transparency, and hence, was mainly used as the base crown for all-ceramic restoration. It could eliminate the aesthetic and biocompatibility problems caused by the metal crown. Long-term clinical observations demonstrated that the decorative ceramic on the surface of zirconium is prone to fracture and chipping during clinical service [3]. This could be attributed to the low compression strength (100–150 MPa) of the decorative ceramic; besides, the linear expansion coefficient between the decorative ceramic and Y-TZP ceramic is different, resulting in low bonding strength [4]. In recent years, with the development of Y-TZP ceramic, the transparent Y-TZP ceramic with specific transparency has begun to be applied in clinical practice [5].The restoration is referred to as monolithic zirconia crown and multiunit fixed dental prosthesis. The problems of decorative ceramic to fracture and chipping can be resolved. Additionally, the monolithic zirconia crown and the multiunit fixed dental prosthesis can be thinned, and the amount of abutment tooth preparation lessens [6–8].

However, the high hardness of Y-TZP ceramic has gained increasing attention to the wear of tooth enamel. Although some *in vitro* studies have shown that the monolithic zirconia crown with the highly polished surface has less wear on tooth enamel and even lower than the Y-TZP ceramic with decorative ceramic [5, 9–11]. To date, a limited number of clinical studies have concentrated on the wearing of the monolithic zirconia crown on natural teeth. The quantitative methods used to measure the wear of the teeth have reported contradictory results [12–16]; thus, it is highly essential to conduct a detailed clinical study on the wear of the monolithic zirconia crown on the antagonist teeth.

In the present study, the following aims were considered: (1) quantitatively study the wear of the monolithic zirconia crown on the antagonist teeth based on the transparent Y-TZP crown, (2) observe the prone position of wear, and (3) explore the possible mechanism of wearing and propose preventive measures to reduce the wear of the antagonist teeth by the monolithic zirconia crown.

## Methods

### Patients

A total of 43 patients (22 men and 21 women), admitted to the Department of Stomatology of the Second Affiliated Hospital of Guilin Medical University (Guilin, China) from May 2018 to November 2018 and needed full-crown restorations for the posterior teeth, were recruited in this study.

The inclusion criteria were as follows: ① The antagonist teeth were natural teeth with normal occlusion and no large filling. The corresponding contralateral teeth were natural teeth without large fillings or crown restorations; ② The results of pulp electrical activity test of abutment before restoration were normal, or perfect root canal therapy is essential before making the crown. The X-ray showed that there was no significant or continuous alveolar bone absorption shadow or absorption less than a third of the root length in the root apex, and also, no fistula and tooth percussion pain was experienced; ③ The periodontal condition of the selected patients was good, and the abutment was not loose; ④ The patients were informed and volunteered to participate in the experiment.

The exclusion criteria were as follows: ① Those who are unable to visit again on time during the observation period; ② Patients with temporomandibular joint disorder syndrome and bruxism, calcium metabolism disorders and osteoporosis, and acute and chronic periodontitis; ③ Patients with malignant tumors of the head and neck with a history of radiotherapy and diabetes, whose blood sugar levels are not stable in the normal range; ④ Pregnant women; ⑤ Those diagnosed or are suspected to have malignant tumors.

This study was approved by the Ethics Committee of the Second Affiliated Hospital of Guilin Medical University (ChiCTR- EPC- 14,005,118).

### Clinical procedures

The abutment teeth were prepared for zirconia crowns according to the tooth preparation guidelines for the zirconia crown [17]. The occlusal reduction was 1.5–2.0 mm, the axial tooth reduction was 1.5–2.0 mm, and the axial wall taper was 6°–8°. The 360° gingival shoulder was shallow and concave-shaped with a slope. After preparing the tooth for the routine gingival retraction (Sure Dental Inc., Seoul, South Korea), it was attempted to tray into the patient’s mouth for the best fit and then coated with DMG Tray Adhesive (DMG Inc., Munich, Germany). Subsequently, the impressions were obtained by the use of a one-step, dual-viscosity technique (Addition silicone rubber, Zhermac Inc., Milan, Italy), and the cast of gypsum material (Die-Stone, Kulzer, LLC., Indiana, USA) was made. The crown shade was selected according to the patient’s choice and the color of the surrounding teeth. The teeth model was sent to the technician center for the monolithic crown fabrication. The crowns were made of yttrium-stabilized zirconia (Zenostar Zr Translucent, Wieland Dental Company, Germany) is a 3Y-TZP. The procedures for crown fabrication included the design of the dental restoration using 3shape CAD Design software (3Shape Inc.,Copenhagen, Denmark), scanning (InEos X5 3D scanner; Dentsply Sirona Inc., Berlin, Germany), cutting (5-axis NC cutting machine; Wieland Dental Co. Ltd., Munich, Germany), grinding to desired contours, followed by coloring, sintering (zirconium oxide sintering furnace; Sinosteel Inc., China), and polishing using ceramic polishers impregnated with diamond abrasives (Shofu Dura Polish Dia, Shofu Dental Co. Ltd., Tokyo, Japan). Polished zirconia has been reported to be more wear-resistant and to cause less wear to enamel antagonists when compared to glazed zirconia in recent studies [13, 18, 19]. Glazed layers can reportedly become worn within 6 months of the restoration [20]. On the other hand, the polishing procedure does not add any layer to the surface of the monolithic zirconia restoration. Moreover, this method can produce a surface roughness of 0.2 µm, which less than or equal to that achieved with glazing [21]. Surface roughness ≤ 0.2 µm provides minimal plaque accumulation and comfortable tactile sensation [22, 23]. Therefore, zirconia polishing may be an effective and time-saving alternative to glazing [2]. Additionally, polishing presented the highest characteristic strength values than heat treatment and glazing after grinding [24].

In the second visit, during the intra-oral try-ins, occlusion and aesthetics of the crowns were checked. The early contact points of the monolithic zirconia crown were marked with occlusion foil (Bausch Articulating Papers Inc., Nashua, NH, USA). The occlusal adjustments were performed with fine-grit diamond burs (TR-13EF, Mani Inc., Tochigi, Japan); the surfaces were subsequently polished thoroughly with the RA322 Polishing Set (EVE Ernst Vetter GmbH, Berlin, Germany) before cementation.

The occlusal contact points were marked intra-orally with 12 µm occlusion foil (Bausch Articulating Papers Inc., Nashua, NH, USA) and photographed after cementation. The participants were advised for adequate oral hygiene and revisited after 6 months and 1 year. After the crowns were cemented with RelyX U100 resin cement in the Clicker Dispenser (3 M Company, Saint Paul, MN, USA), the vinylpolysiloxane impression of the maxillary and mandibular quadrants were made by the secondary impression to locate the monolithic zirconia crowns, their antagonist teeth, the corresponding contralateral teeth, and the antagonist of the corresponding contralateral teeth, and to record the occlusal surfaces of each tooth immediately after cementing of the crowns (baseline) using the addition of silicone rubber (Zhermac Inc.) and again after 6 months and 1 year. The impressions were poured in gypsum material (Die-Stone, Kulzer, LLC., Indiana, USA), and an epoxy resin model (Shanghai Resin Factory Co., Ltd., Shanghai, China) was constructed. The resulting casts were scanned using a 3-dimensional (3D) laser scanner with a precision of 2.1 µm (InEos X5 3D scanner, Dentsply Sirona Inc., Berlin, Germany) to build a digital model. The epoxy resin model was observed under a stereomicroscope (ZOOM-200; Shanghai Caikon Optical Instrument Co., Ltd., Shanghai, China) to determine the parts of natural teeth prone to wear; the microstructure of these parts was observed under a scanning electron microscope (SEM) (S-4800; Hitachi Ltd, Japan).

### Assessment of wearing of the teeth

In order to evaluate the wearing of enamel, it was described in terms of mean vertical loss and mean volume loss of the occlusal contact areas of teeth. In addition to different scanner models, there are also intraoral scanners directly scanning the teeth in the oral cavity to obtain three-dimensional tooth topography data. Although intraoral scanning eliminates the step of casting a plaster model and can theoretically reduce errors, the scanning accuracy of intraoral scanners is relatively low. Vandeweghe et al. [25] found that the maximum scanning accuracy and the accuracy of the oral scanner in vitro were only 30 µm, in addition to the high variability in the results of intraoral scanning [25–29]. Some intraoral scanners need to spray special powder on the tooth surface to increase the scanning error [30]. The thickness of the conventional powder layer is 20–40 µm; however, it might vary according to the operators, thereby reducing the accuracy. Chen et al. [31] studied the scanning accuracy of three intraoral scanners, and their results showed that the range of precision of the three intraoral scanners was 52.38 ± 7.49 to 80.70 ± 15.44 µm. In practical clinical application, many factors may cause additional errors in the accuracy of the scan data. The file of the digital model was imported into Geomagic control 2014.3.0 software (Geomagic Co. Ltd., NC, USA), and the average depth of wearing was calculated through 3D deviation analysis. Then, the digital model was imported into Materialise Magics 22.0 software (Materialise Co., Brussels, Belgium) to calculate the average volume of wearing. The standard deviation of the overlap was required to be < 20 µm, indicating that the errors caused by the alignment overlapping of the digital model were within an acceptable range, thereby providing accurate results [17, 32]. The software had a color scale in mm. The dark blue color indicated the amount of wear. Furthermore, the software-based alignment was conducted through the principle of iterative closest point (ICP) algorithm that was applied for the quantitative assessment of wearing after alignment.

### Statistical analysis

The data were analyzed using SPSS 24.0 software (IBM, Armonk, NY, USA). The vertical and volume loss of the occlusal contact areas of teeth at various follow-up time points are presented as mean ± standard deviation and were analyzed by the Wilcoxon sign rank test. *P* < 0.05 indicated statistical significance.

## Results

### Patients’ demographic and clinical characteristic

The patients were 21–71 (mean, 42.2 ± 13.1)-years-old. A total of 43 teeth, including 16 maxillary first molars, 3 maxillary second molars, 1 maxillary second premolar, 19 mandibular first molars, 3 mandibular second molars, and 1 mandibular second premolar, needed monolithic zirconia crown restorations. Among them, 22 monolithic zirconia crowns were placed in 22 male patients, and 21 monolithic zirconia crowns were placed in 21 female patients. The 43 patients included in the study were successfully followed-up for 1 year.

### Quantification analysis of wearing

**Fig. 1:**
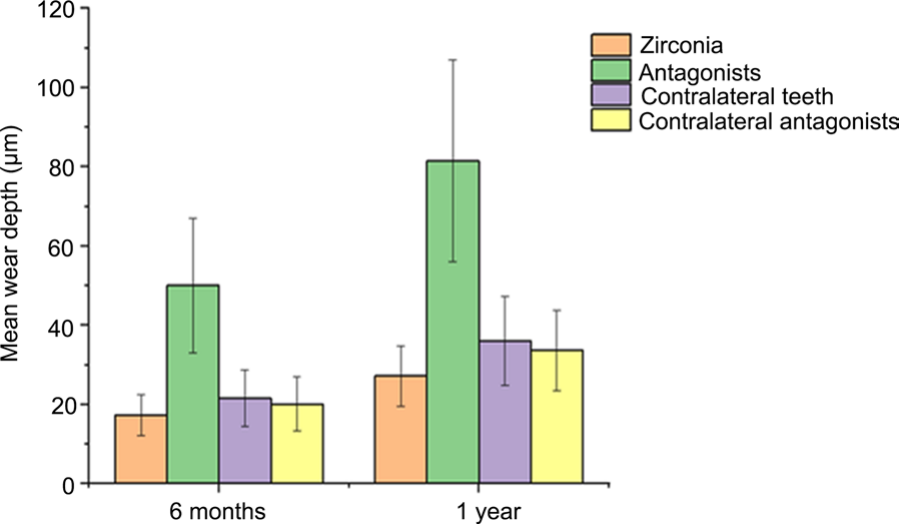
Mean wear depth (µm) of monolithic zirconia crowns and their enamel antagonists and of contralateral teeth and their antagonists after 6 months and 1 year. The lines in the bars indicate standard deviation

The mean vertical loss after 6 months was 50.03 ± 17.02 µm for the enamel of the antagonist teeth, whereas that was 21.55 ± 7.12 and 20.13 ± 6.91 µm for the contralateral teeth and antagonist of the contralateral teeth, respectively. For the monolithic zirconia crown, the mean vertical loss was 17.3 ± 5.23 µm (Fig. 1). Strikingly, the mean vertical loss after 1 year was 81.57 ± 25.49 µm for the enamel of the antagonist teeth, whereas that was 36.13 ± 11.23 and 33.69 ± 10.13 µm for the contralateral natural antagonists. The mean vertical loss for the monolithic zirconia crown was 27.2 ± 7.63 µm (Fig. 1). Significant differences were noted among the enamel wearing of the antagonist teeth and monolithic zirconia crowns/contralateral natural antagonists after 6 months and 1 year (all *P* < 0.01), respectively. The mean vertical loss of the monolithic zirconia crown was the lowest in 6 months and 1 year, which was lower than that of the contralateral natural antagonists (*P* = 0.0098; *P* = 0.0001). No significant difference was noted in depth between the contralateral teeth and the antagonist of the contralateral teeth (*P* = 0.3473).

**Fig. 2:**
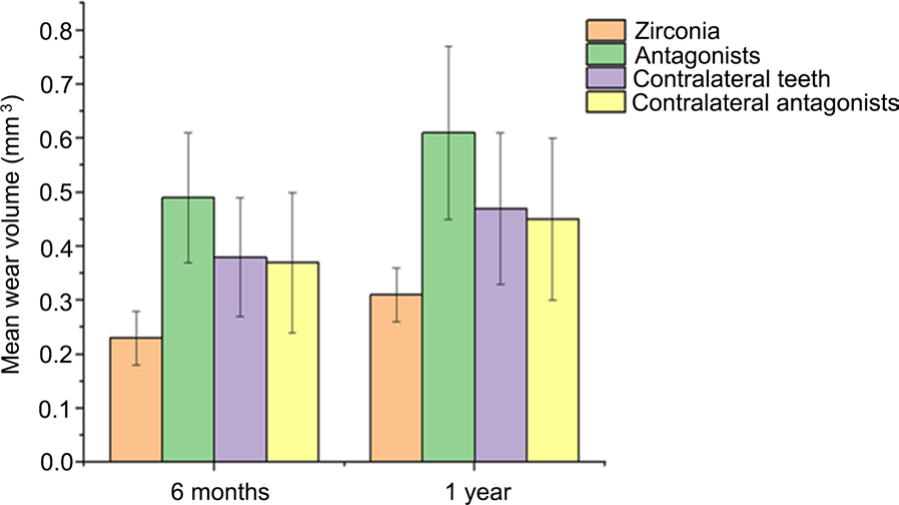
Mean wear volume (mm^3^) of monolithic zirconia crowns and their enamel antagonists and of contralateral teeth and their antagonists after 6 months and 1 year. The lines in the bars indicate standard deviation

Furthermore, with respect to the volume of wearing, the mean volume of wearing of the monolithic zirconia crown, the antagonist teeth, the contralateral teeth, and the antagonist of the contralateral teeth was 0.23 ± 0.05, 0.49 ± 0.12, 0.38 ± 0.11, and 0.37 ± 0.13 mm^3^, respectively (Fig. 2). The mean volume of wearing of the monolithic zirconia crown was significantly smaller than that of the antagonist teeth, the corresponding contralateral teeth, and the antagonist of the contralateral teeth (*P* < 0.0001). On the other hand, the volume of wearing of the antagonist teeth was significantly larger than that of the opposite natural teeth (*P* < 0.0001), and no significant difference was observed in the volume between the contralateral teeth and their antagonists (*P =* 0.6996). After 1 year, the mean volume of wearing of the monolithic zirconia crown, the antagonist teeth, the contralateral teeth, and the antagonist of the contralateral teeth was 0.31 ± 0.05, 0.61 ± 0.16, 0.47 ± 0.14, and 0.45 ± 0.15 mm^3^, respectively (Fig. 2). The mean volume of wearing of the monolithic zirconia crown was significantly smaller than that of the antagonist teeth, the contralateral teeth, and the antagonist of the contralateral teeth (*P* < 0.0001). The mean volume of wearing of the antagonist teeth was markedly larger than that of the natural teeth (*P* < 0.0001), and no significant difference was detected in the volume of wearing between the natural teeth and the antagonist teeth (*P* = 0.5220). The amount of wearing after 1 year was significantly higher than that after 6 months (all *P* < 0.01).

**Fig. 3:**
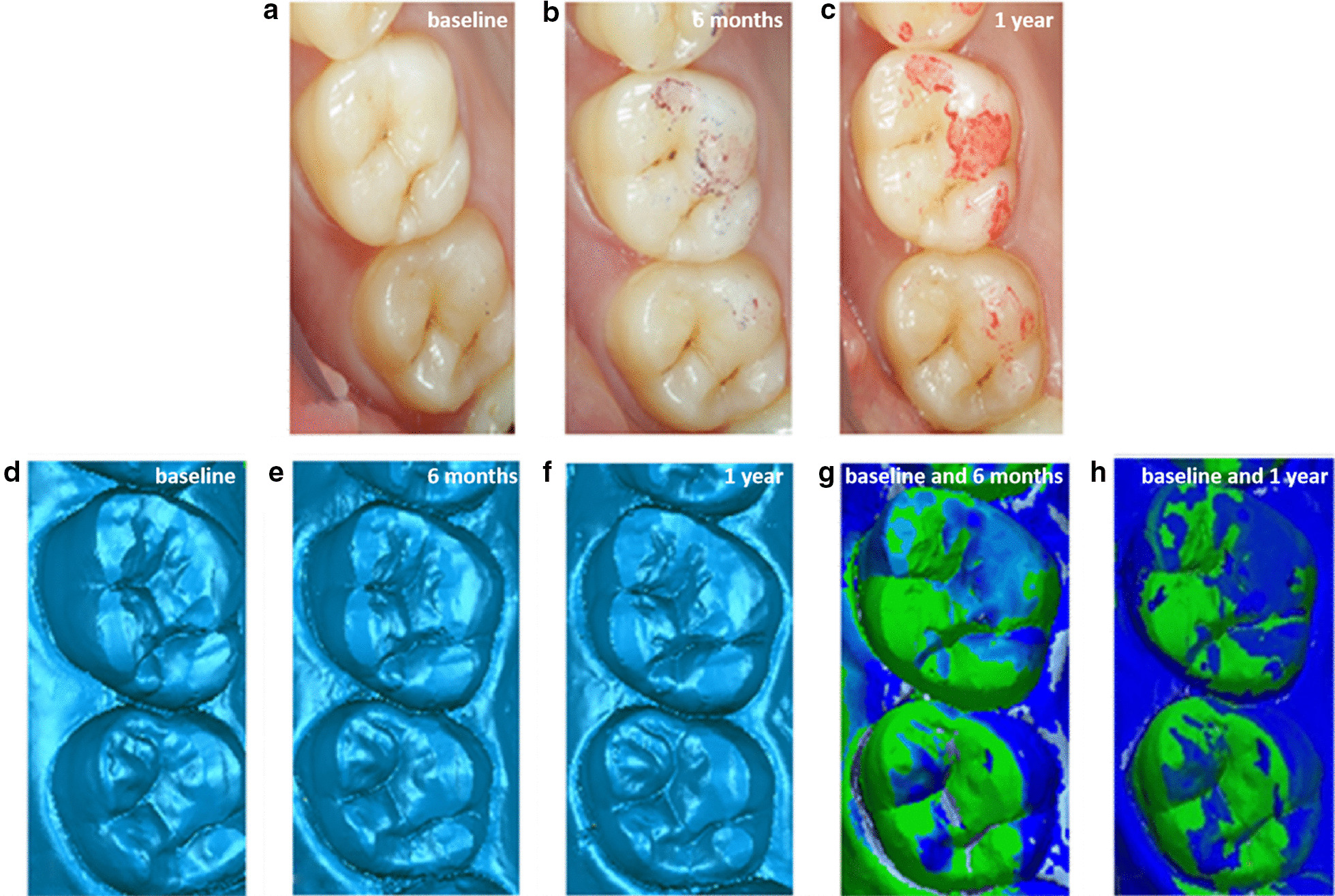
Image of different time intraoral photograph and three-dimensional morphology and overlap deviation of #16 (antagonist): **a** immediately after cementing of the crowns; **b** at the 6-month recall; **c** at the 1-year recall; **d** three-dimensional morphology immediately after cementing of the crowns; **e** three-dimensional morphology at the 6-month recall; **f** dark blue indicates three-dimensional morphology at the 1-year recall; **g** three-dimensional morphology immediately and at 6-month superimposed image showing wear areas; **h** dark blue indicates three-dimensional morphology immediately and at the 1-year superimposed image showing wear areas

As shown in Fig. 3a, b, and c, the markers of the antagonist tooth could be observed at 6 months and 1 year. It was revealed that after 6 months and 1 year, the occlusal contact area was mainly located at the lingual cusp and inclined plane of the teeth. Besides, 3D color deviation was illustrated after immediately overlapping the three-dimensional morphology at 6 months and 1 year. Additionally, the decrease of the height of the occlusal surface was noted after 6 months and 1 year (Fig. 3 g and Fig. 3 h). Moreover, the occlusal contact area was located at the lingual cusp and inclined plane of the teeth. The dark blue parts in Fig. 3 g and h represent the area where, i.e., the area of the worn tooth. The darker the color, the deeper the wear depth. Importantly, the worn parts were mainly located at the lingual cusp and buccal inclined plane of teeth and the lingually inclined plane of the buccal cusp of teeth. Compared with Fig. 3 g, the range of waring on the occlusal surface was enlarged, and the color was deepened after 1 year (Fig. 3 h), indicating that the range and depth of wearing increased.

### Observation of the morphology of worn occlusal surface of the antagonist teeth

**Fig. 4:**
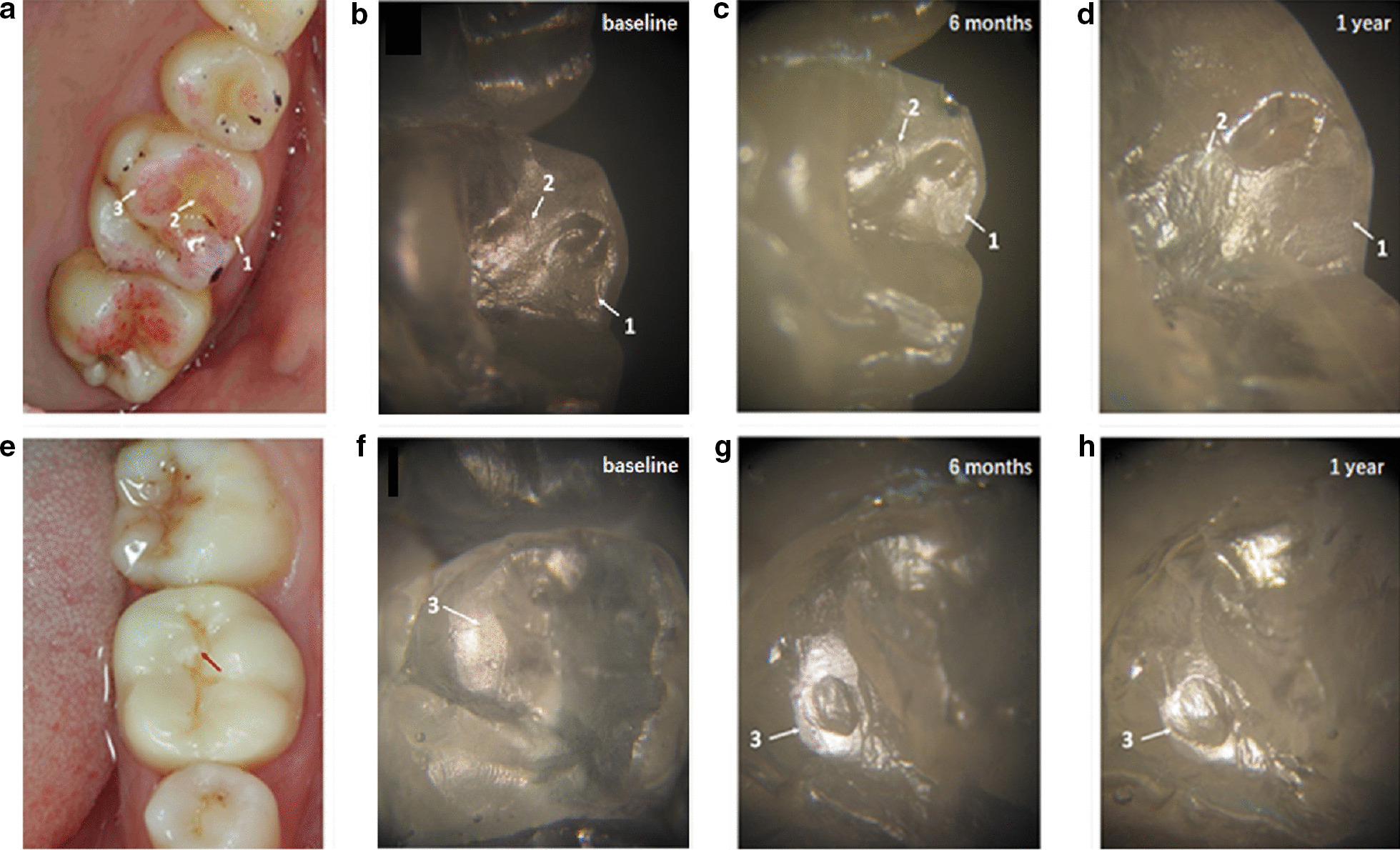
Image of the monolithic zirconia crown on the wear of antagonist teeth a 32-year-old male patient with 26# chronic pulpitis: **a** photo of the occlusal marking of the antagonist teeth (#26); **b** photomicrograph of baseline resin model at arrows 1 and 2 in (**a**); **c** 6-month resin model at arrows 1 and 2 in (**a**), and the wear plane is displayed; **d** 1-year resin model at arrows 1 and 2 in (**a**) shows increased wear plane; **e** the arrow points to the center cusp in the monolithic zirconia crown; **f** baseline resin model at arrows 3 in (**a**); **g** 6-month resin model at arrows 3 in (**a**) and oval wear pits appear; **h** 1-year resin model at arrows 3 in (**a**) and enlarged oval pit

Figure 4 is a stereoscopic micrograph of the epoxy resin model of the antagonist teeth at different observational periods. Figure 4a shows an occlusal marker of the antagonist teeth (#26), including three obvious wears, marked with arrows 1, 2, and 3, respectively. The wear is mainly located at the lingual cusps, with the inclined plane of the teeth and the lingually inclined plane of the buccal cusps of the teeth, which are the working cusps and inclined plane of the teeth, as well as the occlusal contact area. Figure 4b illustrates the proximal buccal cusp of the teeth immediately after the restoration. Additionally, it could be observed that the wearing of the distal inclined plane (arrow 1) and the inclined ridge (arrow 2) of the lingual side of the buccal cusp was not obvious. After 6 months, the distal inclined plane of the lingual side of the buccal cusp formed the wore plane (arrow 1 in Fig. 4c), and the inclined ridge shows distinct wear (arrow 2 in Fig. 4c). After 1 year, the wearing of the distal inclined plane on the lingual side of the buccal cusp was enlarged (arrow 1 in Fig. 4d), and the wearing of the inclined ridge was also enlarged with obvious striated marks; arrow 2 in Fig. 4d displays that the wearing of the monolithic zirconia crown on the natural teeth increased over time. Figure 4f illustrates the resin model of the proximal lingual cusp of antagonist teeth after immediate restoration. Thus, a small plane could be observed on the cusp of the tongue. After 6 months, an oval wear pit was detected on the small plane of the cusp of the tongue (Fig. 4f, arrow 3), and after 1 year, the pit was enlarged (Fig. 4 h, arrow 3). The oval wear pit was corresponding to the central cusp of the monolithic zirconia crown, indicating that the central cusp caused wearing, which increased over time.

**Fig. 5:**
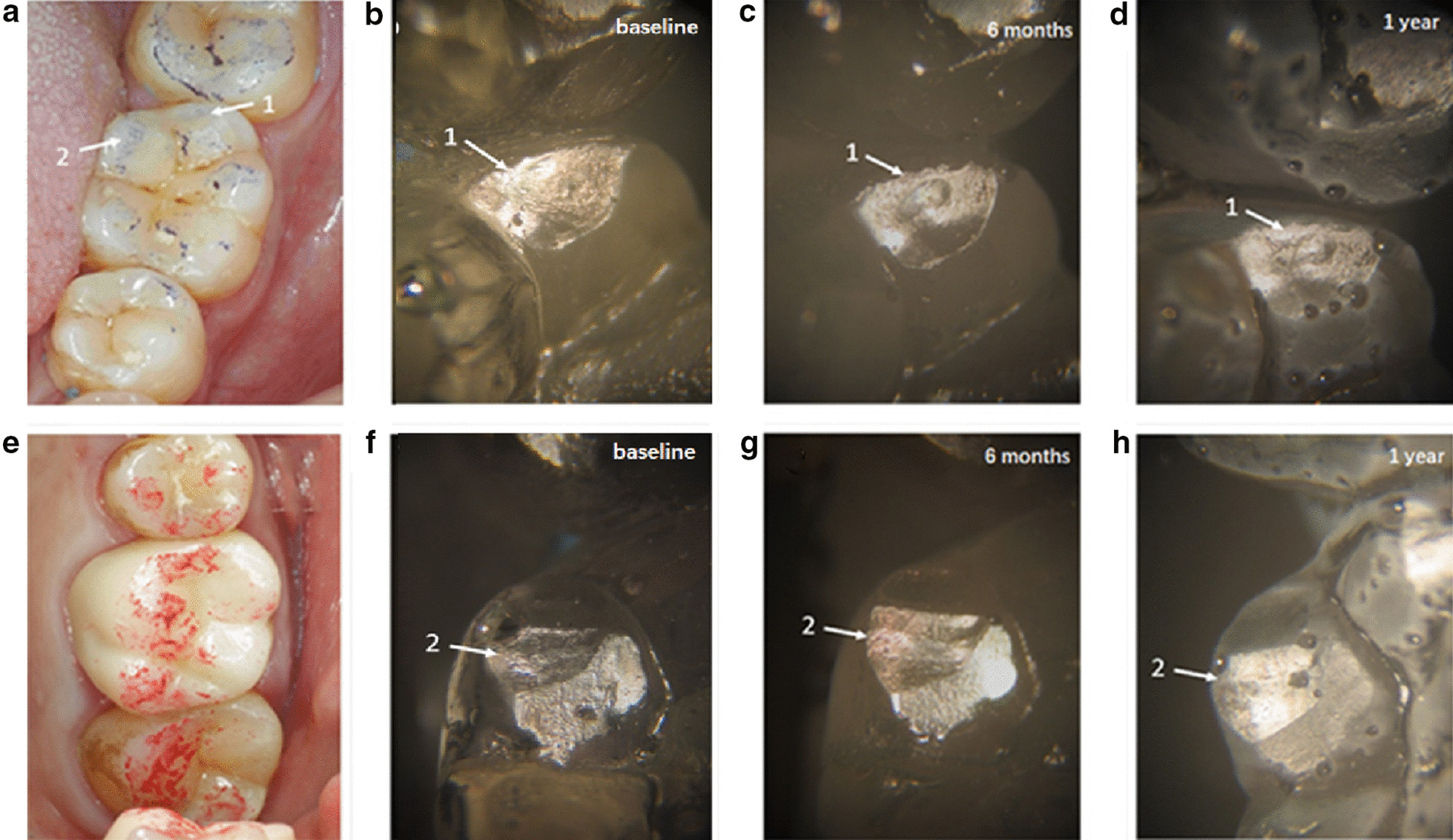
Image of the monolithic zirconia crown on the wear of antagonist teeth in a 21-year-old female patient with the tooth(#36) defect: **a** the photo of the occlusal marking of the antagonist teeth (#36); **b** photomicrograph of baseline resin model at arrows 1 in (**a**); **c** 6-month resin model at arrow 1 in (**a**), the wear pit is displayed; **d** 1-year resin model at arrows 1 in (**a**) show increased wear pit; **e** the monolithic zirconia crown photo; **f** baseline resin model at arrows 2 in **a**; **g** 6-month resin model at arrows 2 in (**a**) and the inclined plane appeared; **h** 1-year resin model at arrows 2 in (**a**) with an enlarged inclined plane

Figure 5 is a stereoscopic micrograph of the epoxy resin model of the antagonist teeth in different observational periods. Figure 5a represents the occlusal marker of the antagonist teeth (#36). Two obvious worn teeth were observed, marked with arrows 1 and 2, respectively. The wear was located at the buccal cusps of the teeth and the lingual inclined plane of the buccal cusps of the teeth and the buccal inclined plane of the lingual cusps of the teeth. Figure 4b displays the lingual inclined plane of the distal cusp of the teeth immediately after the restoration without the wear pit. After 6 months, the inclined plane has worn pit (Fig. 4c, arrow 1), and after 1 year, the pit was expanded (Fig. 4d, arrow 1). Figure 4f shows the wear of the proximal inclined plane of the distal tongue cusp of the antagonist teeth immediately after restoration. The area of wear of the inclined plane expanded significantly after 6 months (Fig. 4 g, arrow 2), and the area of wearing of the inclined plane was further expanded after 1 year (Fig. 4 h, arrow 2).

**Fig. 6:**
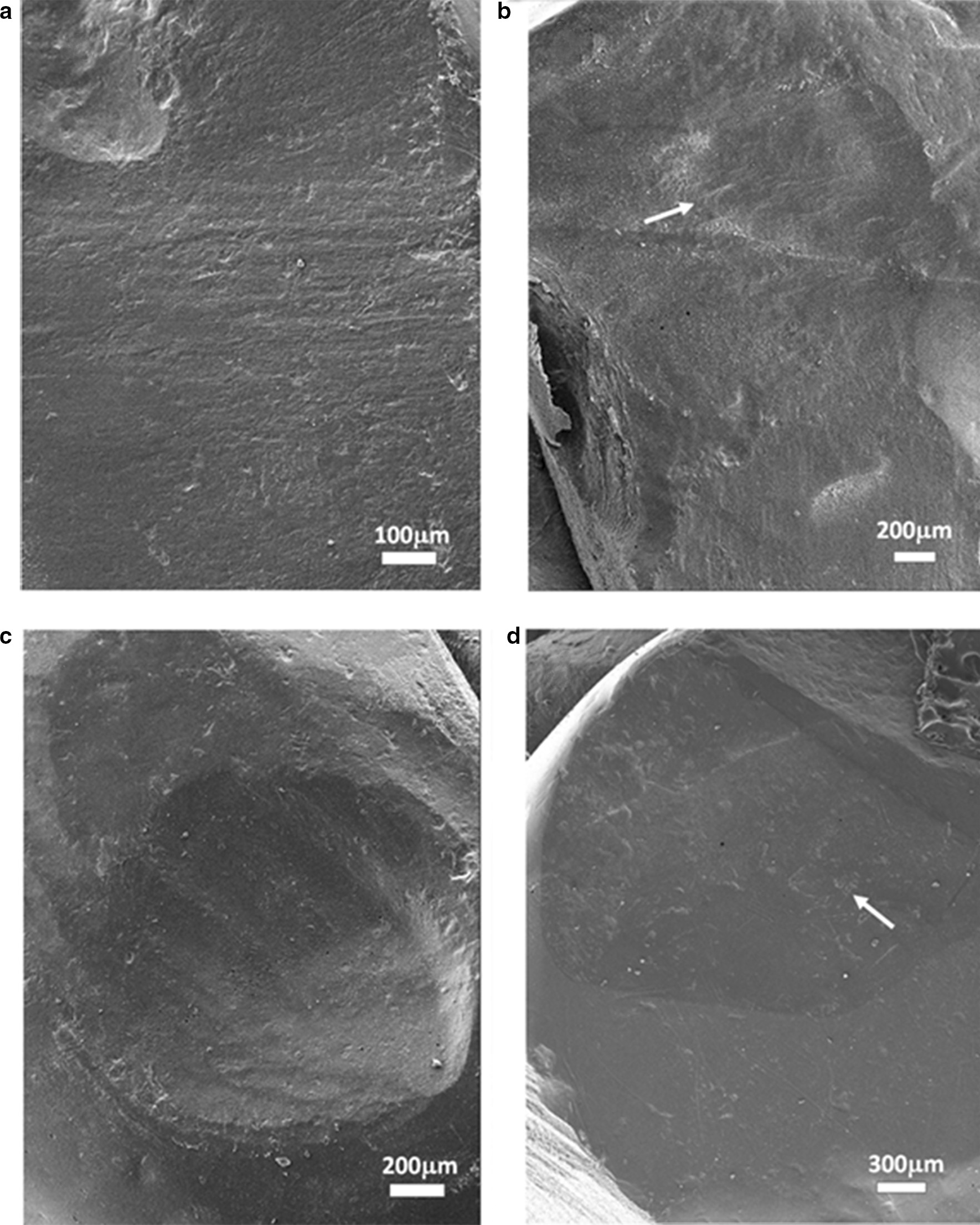
The SEM photographs of the monolithic zirconia crown on the antagonist tooth wear scars: **a** the wear surface indicated by the arrow 1 in (4d), the strip-shaped fine furrows are arranged in parallel, and some small exfoliative pits were scattered; **b** the wearing surface of arrow 2 in (4d), with obvious stripe wear mark (arrow showed); **c** the wear pit indicated by the arrow 3 in (4h). The stripe wear mark on the surface of the pit is clearly visible, with scattered little exfoliated pits on the surface and the edge of the pit; **d** the wearing surface of arrow 2 in (5h). Several small dotted and striped exfoliated pits are observed on the surface

The SEM photographs of the monolithic zirconia crown on the antagonist tooth wear scar are shown in Fig. 6. Figure 6a shows the wear surface indicated by arrow 1 in Fig. 4d; parallel rows of thin stripe furrows with scattered exfoliated pits are observed. Figure 6b is the wearing surface of arrow 2 in Fig. 4d, with stripe wear marks (arrow). Figure 6c is the wear pit indicated by arrow 3 in Fig. 4 h. The stripe wear mark on the surface of the pit is distinct, and there are scattered little exfoliated pits on the surface and the edge of the pit. Figure 6d is the wearing surface of arrow 2 in Fig. 5 h; punctate and striped exfoliated pits are detected on the surface. These photos show that the monolithic zirconia crown’s wear mechanism on the antagonist teeth is mainly abrasive wear and fatigue wear. The cause of stripe wear scars might be the friction of the antagonist teeth enamel caused by the rough structure on the surface of the monolithic zirconia crown, and the little exfoliated pits are typical fatigue wear characteristics.

## Discussion

The current results showed that during the 1-year observation period, the monolithic zirconia crown caused significant wear on the antagonist teeth, which was significantly greater than that between the contralateral natural teeth, while the monolithic zirconia crown caused less self-wear, which was consistent with the results of the study by Stober et al. [17]. In addition, the study showed the cumulative wear amounts (depth and volume) of the monolithic zirconia crown on the natural teeth increased with a prolonged period, and the wear rate of natural teeth was greater in the first half than the second half of the year. This trend of wear growth is consistent with the literature report [12] and the general wear process [33]. Koenig et al. [34] investigated clinical outcomes of second-generation zirconia restorations, including patients with bruxism clinical signs and the material wear process. In that research, complications were observed on antagonistic teeth (3 catastrophic failures). Clinical evaluation of the restorations showed satisfactory results from the aesthetic, functional, and biological perspectives. Besides, zirconia wear was inferior to 15 µm, while glaze wear was observed in all occlusal contact areas after 1 year. In the first half of the year, the natural teeth and the monolithic zirconia crown experienced wear in the run-in period. Figures 4 and 5 show that some protruding cusps and ridge on the occlusal surface of the monolithic zirconia crown have a small contact area with the antagonist teeth at the initial stage of wear, forming the form of point contact. Under the effect of occlusal force, the position where the point of contact with the monolithic zirconia crown occurs on the occlusal surface of the antagonist teeth bears a large bite stress. In this case, the amount of wear is large, which conforms to the general law of wear. As the wear time increases, the contact area increases gradually. As shown in Figs. 4g, h and 6c, the oval pit appeared on the top of the lingual cusp of the antagonist teeth at 6 months due to the wear of the protruding central cusp on the occlusal surface of the monolithic zirconia crown and increased significantly after 1 year. As a result of such wear pits, the contact area with the central cusp of the monolithic zirconia crown increased after the pit was formed, and thus, the compressive stress on the teeth was correspondingly reduced. In this case, both friction and wear processes slowed down gradually. Therefore, the wear of the antagonist teeth in the second half of the year was lower than that in the first half of the year, indicating that the wear between the natural teeth and the monolithic zirconia crown was also self-limited.

There are two main types of wear between teeth: abrasive wear and fatigue wear [35]. The characteristic of abrasive wear is the furrow-shaped wear scars on the wear surface in the same direction and is mainly caused by the rough protrusion of the occlusal surface of the hard restoration in the oral cavity. Due to the high strength, high hardness, and high fracture toughness, the surface of the monolithic zirconia crown is rough with tiny protrusions that will form a file-shaped cutting effect on the antagonist teeth, resulting in wear on the teeth. As shown in Fig. 6a, a large number of parallel strips of furrows can be seen on the wear surface of the antagonist teeth. This could be due to the insufficient polishing effect of the occlusal contact area of the monolithic zirconia crown and the rough surface protrusion that causes abrasion of natural teeth. Several studies are supporting the idea that rough zirconium ceramic restorations will cause greater wear to enamel [13, 14]. Therefore, the adjustment of the monolithic zirconia crown restoration before wearing teeth is critical. During the adjustment, these tiny protrusions on the monolithic zirconia crown occlusal surface should be removed. Another wear mechanism that often occurs between teeth is fatigue wear, which is characterized by chipping and abrasion points or tiny pits on the worn parts of the occlusal surface [33]. Most of the fatigue wear occurs in the parts with a small contact area. Figure 6 shows traces of fatigue wear on several wearing surfaces, mainly the pitted and striped exfoliative pits on the enamel wearing surface, most of which are caused by the protruding cusps and ridges on the monolithic zirconia crown surface to the local contact of the antagonist teeth. The local contact results in considerable compressive stress on the enamel, and the long-term repeated large compressive stress leads to enamel fatigue.

Herein, a fine impression was taken, and the super plaster cast and the epoxy resin model were made. The plaster casts were scanned to establish a digital model of the relevant teeth. The digital model of the teeth in different periods was overlapped by software to quantitatively measure the wear of the occlusal surface of the teeth. Simultaneously, the occlusal surface of the teeth was observed by stereoscopic microscope and SEM to determine the tooth wear sites and the surface microstructure and study the tooth wear qualitatively. In this study, the secondary impression technology of additional silicone rubber accurately replicates the surface morphology and fine structure of teeth. Reportedly [36], the optimized silicone rubber mold-taking technology and the selection of accurate tray-filled plaster casts achieve a linear accuracy of 9 µm, such that they fulfilled the accuracy requirements of the experiment.

In the present study, the three-dimensional morphology overlaps calculation method of wear accurately measured the amount of tooth wear at different time points. Thus, inEos X5 three-dimensional model scanner was used with high scanning accuracy is 2.1 µm, which is less than the normal annual tooth wear depth reported in the literature, so it can meet the needs of this study. In addition to model scanners, intraoral scanners also directly scan the teeth in the oral cavity to obtain three-dimensional tooth topography data. Although intraoral scanning eliminates the step of casting a plaster model and can theoretically reduce errors, the scanning accuracy of intraoral scanners is relatively low. Vandeweghe et al. [25] found that the maximum scanning accuracy and the accuracy of the oral scanner in vitro were only 30 µm in addition to the high variability in the results of intraoral scanning [25–29]. Some intraoral scanners need to spray special powder on the tooth surface to increase the scanning error [30]. The thickness of the conventional powder layer is 20–40 µm; however, it might vary according to the operators [37], thus reducing the accuracy. Thus, it is not appropriate to use an intraoral scanner to study tooth wear.

Thus, the data are accurate [38]. The measurement of the amount of wear on the surface of restoration through three-dimensional topography analysis software facilitates continuous detection and comparison over a prolonged period, and intuitive and quantified wear indicators can be obtained. The three-dimensional images at different time points overlap with the zero-reference plane. After scanning the plaster casts, the scanner generates a digital model and imports it into the three-dimensional topography analysis software to analyze the three-dimensional deviation after overlapping. The standard deviation after overlapping can be controlled within 20 µm, and the error is within the controllable range. The deviation analysis function can perform deviation analysis calculations on the parts that cannot be overlapped (i.e., the worn parts) and obtain accurate quantitative values to evaluate wear.

The published results of in vivo wear experiments are inconsistent. According to Mundhe et al. [16], the mean wear depth of the monolithic zirconia crown on the antagonist teeth after 1 year of use was 127 ± 5.03 µm, and that of the metal ceramic crown on the antagonist teeth was 179.9 ± 8.09 µm. Stober et al. [15, 17] reported the wear results after 6 months as 33 ± 32 µm (antagonist teeth) and 10 ± 5 µm (monolithic zirconia crown), and those after 2 years were 46 ± 30 µm (antagonist teeth) and 14 ± 5 µm (monolithic zirconia crown). This may be due to the small number of their experimental subjects, causing a large difference in the statistical results, experimental detection methods, ethnic characteristics, and eating habits of the subjects. In addition, the patients included in this study were from southern China, and their own dietary habits would affect the experimental results. Although these experimental results are not comparable due to different experimental methods and subjects, it was found that after 1 year, the average wear height of natural teeth in the control group was similar to that of molars, as reported by Mundhe et al. [16]; it was 35.1 ± 2.6 µm, identical to the natural tooth enamel wear of 38 µm/year and the wear stability period of 29 µm/year reported by Lambrechts et al. [39].

Previous studies only assessed the index of wear depth [14–17], and the measurement of the amount of wear was not complete. In addition to using Geomagic Control software to measure the average tooth wear depth, the Boolean operation function of the 3D printing modeling software Materialize Magics was used to measure and calculate the wear volume of the tooth occlusal surface. Boolean operation is a logical operation method of digital symbolization, including union, intersection, and subtraction. When this algorithm is used in graphics processing, a new shape can be generated from a simple combination of basic graphics. This experiment by Materialise Magics Boolean operation functions automatically calculated the two different digital model subtraction part volumes, namely the wear volume at various time points combined with the wear depth, to evaluate the degree of wear comprehensively. The wear volume is a critical indicator to measure the amount of wear because the tooth wear is defined as the volume loss of the tooth tissue [40], and the wear volume reflects the wear degree of the tooth. Some previous studies [39, 41, 42] showed that the wear volume increased proportionally with the extension of time, which was related to the increase in the occlusal contact surface area and the decrease in the occlusal surface height. If only the surface wear depth is used as the main wear parameter index, the data dispersion increases, and the final result is not reliable.

In addition to the quantitative measurement of tooth wear, the epoxy resin model of teeth in different observation periods was observed for the first time by combining stereomicroscope and SEM. The microstructure of tooth wear position and wear surface was studied qualitatively, and the possible mechanism of natural tooth wear caused by a full zirconium crown was discussed. This novel method that combines quantitative and qualitative methods has not been reported previously. It has many advantages to observing the epoxy resin model of teeth in vitro with a stereomicroscope. For example, it can repeatedly and carefully observe each part of the teeth, combine the tooth models of different observation periods for observation and comparison, observe the dynamic changes of the local morphology of the occlusal surface of teeth, and confirm the worn position and the wear situations. In addition, the surface of the epoxy model is compact, and the worn surface is smooth and reflective. Therefore, this method determined many wear positions, wear morphology, and some wear phenomena that are difficult to observe by the naked eye. Next, we observed the surface micromorphology of the determined wear positions under SEM to find the characteristics of the wear surface and infer the putative mechanisms of wear.

Nevertheless, the present study has some limitations. Although the sample size is larger than similar reports, the group wear research is still insufficient, and the observation time is relatively short. Since this study only focused on a single monolithic zirconia crown prosthesis, the wear behavior might differ if large-span fixed dental prostheses are used. Thus, the wear of the monolithic zirconia crown material should also be evaluated in future studies.

## Conclusions

The monolithic zirconia crown has little wear but can cause significant wear to the antagonist teeth. The amount of wear is higher than that between natural teeth in vivo, and it increases with time in a year. The tendency of the wear is to occur at the occlusal contact or early contact points of the monolithic zirconia crown resulting in obvious wear of the antagonist teeth. The wear mechanism of the monolithic zirconia crown on natural teeth is mainly abrasive and fatigue wear.

## Data Availability

The datasets used and/or analysed during the current study available from the corresponding author on reasonable request.

## Abbreviations

SEM: Scanning electron microscope
ICP: Iterative closest point
CAD/CAM: Computer-aided design/computer-aided manufacturing
Y-TZP: Yttria-stabilized tetragonal zirconia polycrystals
